# Pulmonary Function Changes in Fighter Pilots with Positive Pressure Ventilation

**DOI:** 10.3390/healthcare13162020

**Published:** 2025-08-16

**Authors:** Alexander Lengersdorf, Janina Post, Norbert Guettler, Stefan Sammito

**Affiliations:** 1 German Air Force Centre of Aerospace Medicine, 51147 Cologne, Germany; 2 Occupational Medicine, Faculty of Medicine, Otto-von-Guericke-University of Magdeburg, 39106 Magdeburg, Germany

**Keywords:** military, lung function, positive pressure breathing, G-LOC, anti-G suit

## Abstract

**Background/Objectives**: The advancing technological developments of recent decades have also changed the stress profile of pilots of high-performance aircraft (HPA) immensely. Pilots are exposed to different gravitational (G)-forces and are only able to fly with anti-G suits that compensate for the physiological loss of cerebral perfusion by applying external pressure to the body, and positive pressure breathing during G [PBG]. The present study therefore aims to investigate long-term effects of PBG on the lung capacity of fighter pilots. **Methods:** In a retrospective data analysis (1972–2024), the clinical findings of all German military pilots were analyzed. In total, 1838 subjects were included in the analysis, divided into three groups: HPA with PBG, HPA without PBG, and fixed-wing aircraft. **Results:** Lung function analysis showed that no significant decrease in FVC was found in the HPA group with PBG, but a decrease was found in the HPA group without PBG. FEV1 and FEV1/FVC decreased significantly in all groups. Multiple regression analyses indicated that the variables age and aircraft type were significant predictors of the changes in FVC and FEV1, but not for the Tiffeneau index. **Conclusions:** Our study showed that the lung function of HPA pilots who were exposed to both PBG and repeated increased G-forces did not deteriorate to a significantly greater extent compared with other pilots without these conditions; in some cases, it even deteriorated to a lesser extent. Overall, age has primarily been shown to be the predisposing factor for a deterioration in lung function parameters over time.

## 1. Introduction

The advancing technological developments of recent decades have also markedly changed the stress profile of (military) pilots of high-performance aircraft (HPA). While controlling the aircraft used to be the main stress factor, today it is the complex information processing and the physical strain caused by highly agile flight maneuvers.

During these flight maneuvers, (military) pilots are exposed to different gravitational (G)-forces. The accelerations in the head–foot axis (so-called *Z*-axis) are of particular physiological relevance. With the introduction of second-generation fighter aircraft after World War II, the forces acting on the pilot regularly exceeded the natural G-tolerance, which ranges between +3.6 and +5.6 Gz without the use of further anti-G maneuvers [1,2,3,4]. Abruptly occurring Gz acceleration can lead to reduced blood flow to the brain, which can result in almost loss of consciousness (A-LOC) or complete loss of consciousness (G-induced loss of consciousness [G-LOC]). For example, 14.3–43% of the pilots surveyed reported that they had already experienced an A-LOC [5,6,7,8] and 7.7–24% reported experiencing a G-LOC during flight [5,6,9,10,11,12]. Even if the pilots regain consciousness in time, both A-LOC and G-LOC pose considerable risks to flight safety [13].

Compared to their predecessors, the latest generation of modern fighter jets can achieve even higher G-forces and maintain them over longer periods of time. Current aircraft such as the F-22 Raptor, F-35 Lightning II, and the Eurofighter Typhoon are designed for maximum maneuverability and high acceleration capabilities that can generate G-forces exceeding +9 Gz.

In addition to the effects of high Gz forces on blood flow in the brain, they also have an effect on the blood flow and ventilation properties of the lungs. In the lungs, even under normal gravity conditions (+1 Gz), a ventilation–perfusion gradient (ventilation–perfusion ratio, V/Q ratio) is established, wherein the hydrostatic pressure gradient results in better perfusion in the basal lobes of the lungs than apically.

The opposite is true for ventilation. Up to +3 Gz, the total lung capacity (TLC) and vital capacity (VC) are almost constant [14], but the lowering of the diaphragm and of the organs in the thorax stretches the lungs and makes chest expansion more difficult, increasing the functional residual capacity by 300 mL at +2 Gz and by 500 mL at +4 G [14]. At +5 Gz, TLC and VC are already reduced by 15%, which leads to a compensatory increase in respiratory rate [14].

Under increasing Gz loads, the distribution of mechanical forces across the lung becomes non-uniform, resulting in marked changes in the ventilation/perfusion (V/Q) ratio [15]. Functional and model-based studies suggest that this leads to greater expansion and ventilation in apical regions, while basal regions may experience compression, leading to reduced alveolar ventilation and even atelectasis [16]. This leads to the complete enclosure of alveoli, which are no longer ventilated [16,17,18,19]. In addition, increased Gz loads lead to a redistribution of blood to the lower sections of the lungs, so that at +5 Gz, the upper halves of the lungs are hardly supplied with blood at all [15]. As a result, the upper halves of the lungs are well ventilated with no blood flow (V/Q = ∞), while the lower halves of the lungs have a high blood flow but no ventilation (V/Q = 0).

This condition is referred to as acceleration atelectasis in HPA pilots with high Gz loads [20], resulting in a reduced oxygen partial pressure of 56 mmHg at +6 Gz [21] and a 15% reduction in VC [14]. However, the reduction in oxygen supply can be prevented by breathing a higher oxygen concentration [22].

HPA pilots are only able to withstand the described effects of high Gz forces over longer periods of time through the use of anti-G measures. These measures include trainable anti-G straining maneuvers [AGSMs], anti-G suits that compensate for the physiological loss of cerebral perfusion by applying external pressure to the body, and positive pressure breathing during G [PBG] [1,20,23,24]. Also, depending on the HPA aircraft, today there are HPA with PBG (often with the possibility to fly under higher Gz) and HPA without PBG in use.

PBG serves primarily to increase G-tolerance (by up to +2.5 Gz) [24] and significantly reduces the formation of pulmonary atelectasis, which occurs due to a high Gz load, and thus counteracts the minimization of vital capacity [20]. During PBG, high ventilation pressures of up to 70 mmHg can act on the pilot and potentially lead to acute and/or chronic damage to the lung tissue [25]. While there are some studies that have investigated acute effects of PBG in HPA pilots [26], a recently published systematic review [27] identified only two smaller cross-sectional studies [28,29] that looked at long-term effects of PBG. Based on pulmonary function tests on small collectives of fighter jet pilots, they were unable to detect any significant changes compared to the standard values. However, the analyses were only cross-sectional analyses, and so long-term effects could only be determined to a limited extent.

However, the potential chronic effects of positive pressure on the upper airways and lung tissue have not been thoroughly studied. This represents a significant gap in knowledge, given the increasing cumulative exposure of modern pilots to PBG.

The present retrospective study therefore aims to investigate long-term effects of PBG on the lung capacity of fighter pilots in modern combat aircraft. For this purpose, clinical data from periodic medical examinations (PMEs) of military pilots were used and the changes in lung capacity in three differently exposed groups of military pilots over their professional careers were investigated.

## 2. Materials and Methods

In a retrospective data analysis (study period from 1972 to 2024), the clinical findings of all military pilots evaluated at the German Air Force Centre for Aerospace Medicine (GAFCAM) in Cologne were analyzed. The GAFCAM is the central examination center for the PME of all pilots of the German Armed Forces, so that all Air Force, Army, and Navy pilots must be present here at regular intervals (before the age of 40, every three years; after the age of 40, annually). In addition to the PMEs, health records containing the initial medical examination that future pilots had to undergo as part of their application (before starting pilot training, most of them as civilians) were also included in this analysis.

Initially, a database query from the clinical information system identified 11,117 pilots who were eligible for further analysis. Weapon system officers and tactical system officers were also included, as they were exposed to the same G-loads in HPA as the pilot.

Since the clinical information system lacked certain required data, the existing paper files were also used for further data evaluation. In addition to demographic data of the test subjects (gender, age at initial examination as applicant and age at final examination, height, weight, aircraft type flown), the pulmonary function tests from both the initial and final examination were recorded in a Microsoft Access database (Microsoft, Albuquerque, NM, USA). Both spirometry and body plethysmography were used to record lung function parameters. The aim was to isolate three groups of pilots, namely, those who had exclusively flown either HPA with PBG or HPA without PBG, and pilots of fixed-wing aircraft who were used as a control group because they completed their flying duties at similar altitudes but without major Gz loads and without PBG. In a first step, subjects were excluded if their files were no longer available in the archive (*n* = 6249), if their first pulmonary function test was performed after they started flying (*n* = 179), if they did not receive any further pulmonary function tests after the first one and/or if their records did not contain any further flight service data (*n* = 420), or if they were rotorcraft pilots or drone pilots (*n* = 2160). In addition, military pilots in training (*n* = 166), flight instructors (*n* = 21), and subjects who were ill (e.g., had a cold) at the time of the measurement (*n* = 14) were excluded. In total, 1908 pilots could be included with initial and final pulmonary function tests who had flown either an HPA or fixed-wing aircraft.

**Figure 1:**
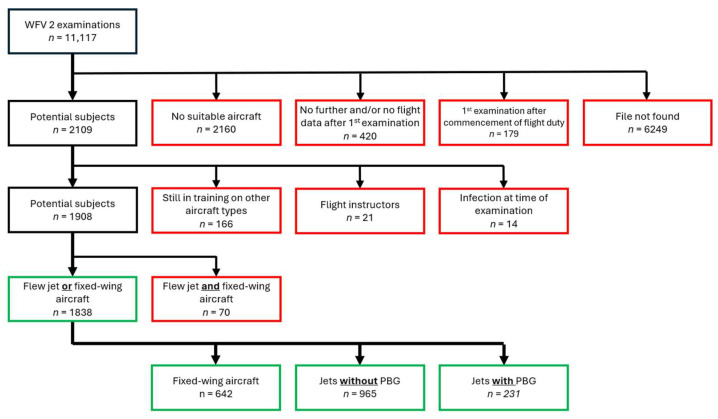
Inclusion and exclusion criteria and categorization of subjects by aircraft type.

Of these test subjects, a further 70 pilots had to be excluded because they had flown both HPA and fixed-wing aircraft in the course of their careers and could therefore not be clearly assigned to one group. This left 1838 pilots for the final evaluation. Of these 1838 pilots, 231 in the “HPA with PBG” group had flown aircraft with PBG, which in Germany is unique to the Eurofighter-2000 “Typhoon” aircraft type. The group “HPA without PBG” comprised 965 persons. They flew aircraft including the following types: Panavia PA-200 “Tornado”, McDonell F-4 Phantom II, Mikoyan Gurevich MiG29, and Alpha Jet A. The control group was formed by pilots of fixed-wing aircraft, with 642 test subjects who flew the aircraft types P-3C “Orion”, Bombardier Challenger 601, and Global 500/600, the Airbus models A310, A319/320/321, A330, A340, and A400M “Atlas”, C-130J “Hercules”, Transall C-160D, Boeing E-3A, DO 228 LM, BR1150 Breguet Atlantic, and other civilian fixed-wing aircraft. Figure 1 provides an overview of the methodological approach.

As part of the data collection, existing lung function tests were used, which were routinely carried out as part of the PME using spirometry or body plethysmography. As the use of body plethysmography was not consistently available, the forced vital capacity [FVC], the forced expiratory volume in the first second [FEV1], and the FEV1/FVC ratio were analyzed in the further analysis of this study.

The examinations from the initial screening (as a pilot candidate before commencing pilot training) and the most recent available examination were compared. In addition, demographic and anthropometric data (age, gender, height, weight) were taken into account. These data serve to identify possible correlations between the individual characteristics of the test subjects and the observed changes in lung function. The recorded variables are based on the difference in lung function values between the initial and final examinations. Data were reviewed for plausibility. Potential outliers suggestive of input errors were rechecked and corrected if necessary. An indication of this was a deviation of measured values from the norm.

Data were collected using Microsoft Access (Microsoft, Albuquerque, NM, USA). After exporting the raw data, statistical analysis was performed with IBM SPSS Statistics 24 (IBM, Armonk, NY, USA) after prior anonymization of the data. Descriptive data analysis was performed using medians and interquartile ranges (IQRs), as well as frequency distributions, due to a lack of normal distribution using Kolmogorov–Smirnov tests. The Wilcoxon test and Kruskal–Wallis test were applied to test for differences between initial and final examinations. Multiple regression analyses were used to examine the influence of external variables such as aircraft type, smoking status, age, and gender on lung function parameters.

This study is part of the departmental research activities of the GAFCAM. No additional medical, diagnostic, or therapeutic measures were carried out for the analysis, and no formal approval was required for the purely retrospective analysis in accordance with the specifications of the responsible ethics committee of the North Rhine Medical Association, Germany. In addition, the responsible data protection officer reviewed the study protocol.

## 3. Results

A total of 1838 subjects were included in the analysis, divided into three groups: HPA with PBG (*n* = 231), HPA without PBG (*n* = 965), and fixed-wing aircraft (*n* = 642). The majority of subjects were male (99%), with a median age of 39.9 years (IQR: 34.8 to 45.9 years). The median follow-up time was 19.7 years (IQR: 14.7 to 25.3 years). Smoking status varied significantly between groups (*p* < 0.001), with a higher proportion of never smokers in the HPA with PBG group (75%) than in the HPA without PBG (67%) and fixed-wing aircraft (63%) groups. There were also significant differences in body length, body weight, and body mass index between the groups (*p* < 0.001). An overview is presented in Table 1.

Lung function analysis showed that no significant decrease in FVC was found in the HPA group with PBG (*p* = 0.123), but in the HPA group without PBG, FVC decreased by a median of 0.03 L (*p* = 0.031), and in the fixed-wing aircraft group, it decreased by a median of 0.14 L (*p* < 0.001).

FEV1 decreased significantly in all groups (*p* < 0.001 in each case). This decrease was greatest in the fixed-wing aircraft group (median 0.54 L/s), in contrast to the HPA group with PBG (0.46 L/s) and the HPA group without PBG (0.35 L/s) The extent of reduction also differed significantly between the groups (*p* < 0.001).

The FEV1/FVC ratio also fell significantly in all groups (*p* < 0.001 in each case), wherein the reduction was greatest in the HPA group with PBG at a median of 8.2%, while it fell by 7.4% in the fixed-wing aircraft group and by 6.0% in the HPA group without PBG. The reduction was also significant between the groups (*p* < 0.001). Although a statistically greater decrease in the FEV1/FVC ratio was observed in the group exposed to PBG, the values remained within the expected physiological range, with no evidence of a clinically significant obstructive pattern. Table 2 presents an overview including the respective IQR.

Multiple function analyses demonstrated that the variables age and aircraft type (HPA with PBG vs. HPA without PBG vs. fixed-wing aircraft) were significant predictors of the changes in FVC (*p* < 0.05) and FEV1, while gender and smoking status had no significant influence (see Table 3 and Table 4).

In contrast, none of the selected variables (smoking status, age, gender, aircraft group) were significant predictors of changes in the Tiffeneau index (*p* > 0.05 in each case, see Table 5).

## 4. Discussion

The present retrospective study, with a median follow-up period of 19.7 years, was able to show that the lung function of HPA pilots who were exposed to both PBG and repeated increased G-forces did not deteriorate to a significantly greater extent compared with other pilots who were only exposed to increased G-forces or compared to fixed-wing pilots; in some cases, it even deteriorated to a lesser extent. Overall, age has primarily been shown to be the predisposing factor for a deterioration in lung function parameters over time, which is in conjunction with the literature [30,31,32,33].

To date, changes in lung function caused by PBG in fighter pilots have only been the focus of a few studies. In a human centrifuge study with three test subjects, acute changes in lung function parameters were examined with and without PBG, and with and without anti-G vests [34]. This resulted in a reduction in total lung capacity and inspiratory capacity, although no differences in vital capacity were observed when the human centrifuge run was performed with or without PBG in addition to the Gz load. In a recently published study in a human centrifuge, no negative, but rather positive, changes in lung function parameters were shown at two sites as a result of isolated Gz exposure up to +9 Gz, indicating increased recruitment due to the anti-G maneuvers [35]. Another study investigated acute changes in lung function in HPA pilots with increased G-loads and the use of PBG during training flights. Bojahr et al. [26] demonstrated that short-term changes in respiratory function and an increase in oxidative stress occurred. However, there have only been a few studies so far that have investigated the long-term effects of PBG on lung function. In a systematic review [27], only two smaller cross-sectional studies were identified [28,29], which reported a potential reduction in specific lung function parameters (FVC, FEV1, FEV1/FVC, PEF, FEF25–75%, and residual volume) in connection with work as an HPA pilot with the simultaneous presence of PBG. Neither study showed any significant major changes in these lung function parameters in the HPA pilots studied. However, it should be noted that the two studies only examined small collectives (21 and 109 HPA pilots, respectively) and were only cross-sectional analyses. To the best of our knowledge, the present study is the first one to investigate possible changes in lung function parameters over such a long period of time.

PBG offers several operational advantages in the context of HPA use. For example, it reduces atelectasis [20,22], reduces pilot fatigue [25,36,37,38], increases G-tolerance [37,39], extends G-tolerance time [25,36], reduces the G-LOC rate [38,40], and is highly accepted by pilots [38]. The disadvantages of PBG, on the other hand, appear to be rather minor. Pilots report a dry cough immediately after use [38].

The findings of our study are consistent with the two previous studies conducted on smaller numbers of subjects [28,29]. Although our study showed a greater decrease in the FEV1/FVC ratio among pilots with PBG, there was no significant impairment of FEV1.

A major strength of the present study is the long follow-up period and the large sample size, which enables a solid statistical analysis. Due to the use of very detailed filter options and access to the electronic and paper-based files at GAFCAM, a very large dataset was available. This ensured that a total sample could be assumed, as all military pilots of the German Armed Forces (Bundeswehr) have to complete a PME at the GAFCAM at regular intervals. This helped minimize selection bias by ensuring comprehensive inclusion of all pilot groups.

In addition, only standardized lung function tests from the medical examination records were used, which ensures a high degree of comparability of the data. As the examinations used all originate from the GAFCAM, with a few exceptions (when external findings had to be used), it can be assumed that the examination procedure remained largely consistent over time.

A further strength of the present study is the division into a group of HPA pilots who were exposed to both increased Gz loads and PBG, a group of HPA pilots who were only exposed to increased Gz loads without PBG, and a control group that was exposed to neither higher Gz loads nor PBG. The results show that neither PBG nor multiple Gz exposures alone were associated with a deterioration in lung function parameters.

Limitations include the focus on only three lung function parameters, which makes a comprehensive assessment of lung function difficult. In particular, there is a lack of measured values for the peak expiratory flow (PEF), which would be relevant for the assessment of obstructive respiratory diseases and which would have required the continuous use of body plethysmography. CO diffusion measurement would also be necessary for the diagnosis of pulmonary emphysema. In the present analysis, only the three parameters mentioned could be used, as previously, only a lung function test was performed, and only in recent years has body plethysmography become the standard method in the context of PME. In the future, care should at least be taken to ensure that a body plethysmography is always carried out as part of the screening in order to be able to better assess the development of obstructive diseases.

In addition, the heterogeneous exposure of the pilots represents a methodological limitation, in particular due to retraining on aircraft types with PBG after previous use without PBG. As the individual duration of exposure to PBG was not recorded in the medical files, it was not possible to precisely differentiate the actual PBG use among the groups. This resulted in subjects with mixed exposure being categorized into the HPA with PBG group, making it difficult to analyze the direct effects of PBG. However, this cannot be completely avoided, as every pilot is first trained for a certain period of time on aircraft that do not have PBG and then switches to the highly dynamic aircraft types.

It must also be taken into account that military pilots who, due to a lung disease that could potentially be caused by PBG, have an inability to fly and thus retire from active service would not be further examined in the context of military pilot fitness. This means that follow-up studies would only be available to a limited extent. However, this distortion of the data situation, known as the healthy worker effect [41], can be ruled out for the present study, as the full coverage over a very long period (1972–2024) means that possible pilots in which this effect would have occurred would have been noticed during data collection. Only one pilot was permanently disqualified from flying due to a lung disease, which was caused by a malignant tumor. It should be noted that pilots who fly HPA are usually highly selected personnel in very good medical condition at the start of their military training. Potential candidates who are already suffering from respiratory diseases, like COPD, asthma, etc. (i.e., before joining the military), are classified as unfit for duty as a pilot (moreover, they are generally not fit for military service either).

## 5. Conclusions

In summary, the results of this long-term study show that despite the high mechanical stress on the airways due to overpressure and increased Gz loads, pulmonary adaptation mechanisms do not appear to have a clinically relevant adverse effect over time, and PBG thus remains an effective and safe measure to increase Gz tolerance.

## Figures and Tables

**Table 1:** Comparison of demographic and clinical characteristics (gender, age, follow-up time, smoking status, body weight, body height, and body mass index [BMI]) between the three study groups and the overall group; HPA = high-performance aircraft; PBG = positive pressure breathing.

	HPA with PBG	HPA Without PBG	Fixed-Wing Aircraft	Total	*p*_group_
***n***	231 (100%)	965 (100%)	642 (100%)	1838 (100%)	
**Gender**					0.765
Male	229 (99%)	959 (99%)	636 (99%)	1824 (99%)	
Female	2 (1%)	6 (1%)	6 (1%)	14 (1%)	
**Age [years]**	37.9 (33.0–43.9)	40.0 (36.9–43.0)	39.8 (40.5–49.2)	39.9 (34.8–45.9)	**0.002**
**Follow-up [years]**	18.1 (14.0–24.8)	19.9 (16.8–23.2)	19.2 (12.6–28.4)	19.7 (14.7–25.3)	**0.032**
**Smoking status**					**<0.001**
Active smoker	25 (11%)	160 (17%)	82 (13%)	267 (15%)	
Former smoker	28 (12%)	138 (14%)	150 (23%)	316 (17%)	
Never smoked	173 (75%)	644 (67%)	404 (63%)	1221 (66%)	
Not specified	5 (2%)	23 (2%)	6 (1%)	34 (2%)	
**Body weight [kg]**					
Initial	72.0 (68.0–78.0)	72.0 (67.0–76.3)	74.0 (68.2–80.0)	72.1 (68.0–78.0)	**<0.001**
Last	81.7 (76.0–89.0)	82.0 (76.0–88.0)	85.0 (78.0–92.1)	83.0 (76.1–90.0)	**<0.001**
Difference	9.9 (5.0–13.1)	10.0 (5.6–15.0)	10.0 (5.5–15.0)	10.0 (5.5–15.0)	0.329
*p*_initial vs. last_	**<0.001**	**<0.001**	**<0.001**	**<0.001**	
**Body height [cm]**					
Initial	180 (177–184)	180 (176–184)	182 (178–186)	181 (177–185)	**<0.001**
Last	181 (177–185)	181 (177–185)	183 (178–187)	182 (178–186)	**<0.001**
Difference	−1 (−1–0)	−1 (−2–0)	−1 (−2–0)	−1 (−2–0)	0.168
*p*_initial vs. last_	**<0.001**	**<0.001**	**<0.001**	**<0.001**	
**BMI [kg/m^2^]**					
Initial	22.2 (21.1–23.9)	22.1 (21.0–23.3)	22.4 (21.0–23.8)	22.2 (21.0–23.5)	**0.013**
Last	24.8 (23.5–26.6)	24.9 (23.5–26.5)	25.2 (23.7–27.5)	25.1 (23.6–26.9)	**0.012**
Difference	2.7 (1.5–3.9)	2.8 (1.5–4.3)	2.8 (1.5–4.4)	2.8 (1.5–4.3)	0.520
*p*_initial vs. Last_	**<0.001**	**<0.001**	**<0.001**	**<0.001**	

**Table 2:** Comparison of lung function parameters (FVC, FEV1, and FEV1/FVC) between the three study groups and the overall group; HPA = high-performance aircraft; PBG = positive pressure breathing.

	HPA with PBG	HPA Without PBG	Fixed-Wing Aircraft	Total	*p*_group_
***n***	231 (100%)	965 (100%)	642 (100%)	1838 (100%)	
**FVC [L]**					
Initial	5.41 (5.04–5.85)	5.48 (5.07–5.97)	5.53 (5.09–6.07)	5.50 (5.07–6.00)	**0.025**
Last	5.34 (4.99–5.87)	5.42 (4.95–5.89)	5.41 (4.92–5.97)	5.41 (4.96–5.91)	0.959
Difference	−0.05 (−0.36–0.28)	−0.03 (−0.40–0.33)	−0.14 (−0.52–0.24)	−0.07 (−0.44–0.30)	**<0.001**
*p*_initial vs. last_	**0.123**	**0.031**	**<0.001**	**<0.001**	
**FEV1 [L/s]**					
Initial	4.67 (4.34–5.10)	4.67 (4.30–5.02)	4.74 (4.37–5.18)	4.69 (4.33–5.08)	**0.010**
Last	4.25 (3.86–4.60)	4.31 (3.88–4.72)	4.23 (3.77–4.71)	4.28 (3.84–4.70)	0.124
Difference	−0.46 (−0.75–−0.22)	−0.35 (−0.68–−0.03)	−0.54 (−0.86–−0.18)	−0.45 (−0.76–−0.12)	**<0.001**
*p*_initial vs. last_	**<0.001**	**<0.001**	**<0.001**	**<0.001**	
**FEV1/FVC [%]**					
Initial	87.0 (83.0–91.3)	85.0 (81.0–90.0)	85.3 (82.0–90.9)	85.6 (82.0–90.0)	**0.005**
Last	78.9 (74.9–82.5)	79.8 (75.7–83.6)	78.7 (74.4–82.4)	79.3 (75.0–83.1)	**0.001**
Difference	−8.2 (−11.8–−5.0)	−6.0 (−10.0–−2.2)	−7.4 (−11.4–−3.6)	−6.8 (−10.7–−3.0)	**<0.001**
*p*_initial vs. last_	**<0.001**	**<0.001**	**<0.001**	**<0.001**	

**Table 3:** Multiple regression analysis: influence of selected variables on the change in forced vital capacity (FVC).

	*b*	SE *b*	β	T	*p*
(Constant)	1.354	0.170		7.950	**<0.001**
Smoking status	0.000	0.001	−0.007	−0.328	0.743
Age	−0.029	0.002	−0.397	−18.389	**<0.001**
Gender	−0.159	0.146	−0.023	−1.087	0.277
Aircraft group	−0.039	0.019	−0.043	−2.016	**0.044**

*n* = 1838; R^2^ = 0.161; corrected R^2^ = 0.160; F (4, 1833) = 88.220; *p* < 0.001.

**Table 4:** Multiple regression analysis: influence of selected variables on the change in forced expiratory volume in one second (FEV1).

	*b*	SE *b*	β	T	*p*
(Constant)	0.602	0.161		3.751	**<0.001**
Smoking status	0.000	0.001	−0.004	−0.193	0.847
Age	−0.023	0.001	−0.343	−15.511	**<0.001**
Gender	−0.014	0.138	−0.002	−0.099	0.921
Aircraft group	−0.039	0.018	−0.046	−2.102	**0.036**

*n* = 1838; R^2^ = 0.122; corrected R^2^ = 0.120; F (4, 1833) = 63.777; *p* < 0.001.

**Table 5:** Multiple regression analysis: influence of selected variables on the change in the Tiffeneau index (FEV1/FVC).

	*b*	SE *b*	β	T	*p*
(Constant)	−7.196	1.898		−3.791	**<0.001**
Smoking status	−0.008	0.011	−0.018	−0.774	0.439
Age	−0.005	0.018	−0.007	−0.288	0.774
Gender	0.576	1.628	0.008	0.353	0.724
Aircraft group	−0.092	0.217	−0.010	−0.425	0.671

*n* = 1838; R^2^ = 0.001; corrected R^2^ = −0.002; F (4, 1833) = 0.257; *p* = 0.905.

## Data Availability

The analyzed datasets are property of the German Armed Forces (Bundeswehr). They are available upon reasonable request.

## Abbreviations

The following abbreviations are used in this manuscript:

AGSM	anti-G straining maneuvers
A-LOC	almost loss of consciousness
FEV1	forced expiratory volume in the first second
FVC	forced vital capacity
GAFCAM	German Air Force Centre for Aerospace Medicine
G-LOC	G-induced loss of consciousness
HPA	high-performance aircraft
IQR	interquartile range
PBG	positive pressure breathing during G
PEF	peak expiratory flow
PME	periodic medical examination
TLC	total lung capacity
V/Q	ventilation/perfusion
VC	vital capacity
